# Matching an Old Marine Paradigm: Limitless Connectivity in a Deep-Water Fish over a Large Distance

**DOI:** 10.3390/ani13172691

**Published:** 2023-08-23

**Authors:** Alice Ferrari, Martina Spiga, Miriam Dominguez Rodriguez, Fabio Fiorentino, Juan Gil-Herrera, Pilar Hernandez, Manuel Hidalgo, Carolina Johnstone, Sana Khemiri, Kenza Mokhtar-Jamaï, Irene Nadal, Montse Pérez, Simone Sammartino, Marcelo Vasconcellos, Alessia Cariani

**Affiliations:** 1 Department of Biological, Geological & Environmental Sciences (BiGeA), University of Bologna, 40126 Bologna, Italy; martina.spiga5@unibo.it (M.S.); alessia.cariani@unibo.it (A.C.); 2 Centro Oceanográfico de Málaga (IEO, CSIC), 29640 Málaga, Spain; mdr8625@gmail.com (M.D.R.); carolina.johnstone@ieo.csic.es (C.J.); 3 Institute for Marine Biological Resources and Biotechnology (IRBIM), National Research Council (CNR), 91026 Trapani, Italy; fabio.fiorentino@irbim.cnr.it; 4 Stazione Zoologica Anton Dorhn, 90149 Palermo, Italy; 5 Centro Oceanográfico de Cádiz (IEO, CSIC), 11006 Cádiz, Spain; juan.gil@ieo.csic.es; 6 Technical Unit for Western Mediterranean, General Fisheries Commission for the Mediterranean (GFCM), Fisheries and Aquaculture Division, Food and Agricultural Organization (FAO) of the United Nations, 29014 Malaga, Spain; pilar.hernandez@fao.org; 7 Oceanographic Center of the Balearic Islands, Ecosystem Oceanography Group (GRECO), Spanish Institute of Oceanography (IEO, CSIC), 07015 Palma, Spain; jm.hidalgo@ieo.csic.es; 8 Institut National des Sciences et Technologies de la Mer, Salammbô 2025, Tunisia; sanak182000@yahoo.com; 9 Laboratoire de Génétique des Populations Halieutiques, Institut National de Recherche Halieutique (INRH), Centre Régional d’Agadir, Agadir 80000, Morocco; mokhtarjamai@inrh.ma; 10 Physical Oceanography Group, Instituto de Biotecnología y Desarrollo Azul (IBYDA), Universidad de Málaga, 29071 Málaga, Spain; irenenadal@ctima.uma.es; 11 AquaCOV, Centro Oceanográfico de Vigo (IEO, CSIC), 36390 Pontevedra, Spain; montse.perez@ieo.csic.es; 12 Physical Oceanography Group, Instituto de Ingeniería Oceánica (IIO), Universidad de Málaga, 29071 Málaga, Spain; ssammartino@ctima.uma.es; 13 Fisheries and Aquaculture Division, Food and Agriculture Organization (FAO) of the United Nations, 00153 Rome, Italy; marcelo.vasconcellos@fao.org

**Keywords:** population structure, microsatellite, connectivity, Blackspot Seabream, *Pagellus bogaraveo*, fishery resource

## Abstract

**Simple Summary:**

The response of marine fish species to external pressures highly depends on their intrinsic bio-ecological traits. Among those species of commercial interest, the deep-water Blackspot Seabream (*Pagellus bogaraveo*, Brünnich 1768) inhabits a large geographical range, a condition that might contribute to high resilience to fishing activity. The biology of the species has been patchily investigated in past years, and to date a complete picture of its connectivity across its distribution area (Eastern Atlantic Ocean and Mediterranean Sea) has not been available. We investigated the species’ genetic variability and differentiation at a very large geographical scale by analysing nuclear DNA markers. The absence of genetic population structuring over such a wide area was found, strengthening the hypothesis that egg and larval dispersal are fundamental in sustaining the genetic connectivity of the Blackspot Seabream.

**Abstract:**

Investigations of population structuring in wild species are fundamental to complete the bigger picture defining their ecological and biological roles in the marine realm, to estimate their recovery capacity triggered by human disturbance and implement more efficient management strategies for fishery resources. The Blackspot Seabream (*Pagellus bogaraveo*, Brünnich 1768) is a commercially valuable deep-water fish highly exploited over past decades. Considering its exploitation status, deepening the knowledge of intraspecific variability, genetic diversity, and differentiation using high-performing molecular markers is considered an important step for a more effective stock assessment and fishery management. With one of the largest efforts conceived of and completed by countries overlooking the Atlantic and Mediterranean coasts in recent years, a total of 320 individuals were collected from different fishing grounds in the Mediterranean Sea and Atlantic Ocean and analysed using 29 microsatellite loci. We applied multiple statistical approaches to investigate the species’ connectivity and population structure across most of its described distribution area. Considering the incomplete knowledge regarding the migratory behaviour of adults, here we suggest the importance of egg and larval dispersal in sustaining the observed genetic connectivity on such a large geographical scale.

## 1. Introduction

Genetic structuring in marine populations has been demonstrated to be the result of historical and contemporary interactions among complex scenarios involving bio-ecological, demographic, behavioural, evolutionary, and oceanographic processes [1,2,3]. The latter factors can affect species at very different life stages, determining rates and patterns of dispersal of gametes, zygotes, and larvae, representing the phases with the highest movement opportunities, and adults. In addition, stochastic and deterministic forces (i.e., genetic drift, natural selection) are intrinsically bound to the survival and reproductive success of individuals and can contribute to spatial and temporal connectivity within and among populations [4]. In recent years, an increasing number of case studies of marine species (i.e., otoliths, parasites, tagging [5,6,7]), conducted combining different approaches, demonstrated that local adaptation and/or oceanographic barriers can maintain genetic divergence between geographical populations [8,9,10]. The presence or absence of fragmented patterns of genetic variation should thus be considered for conservation and management purposes in order to avoid the erosion of genetic adaptive potential. From this perspective, maintaining high levels of diversity within species and understanding their response to human disturbance (e.g., habitat fragmentation, climate change, and overexploitation) are equally important, especially when looking at commercially appreciated wild species [11].

Among Sparidae, the Blackspot Seabream (*Pagellus bogaraveo*, Brünnich 1768) is a large-sized, benthopelagic species common in the Eastern Atlantic Ocean from Norway and Sweden, to Cape Blanc (Morocco), Madeira, Canary, and Azores Islands, as well as in the western and central Mediterranean Sea and the Aegean Sea [12,13,14,15]. This species lives in small shoals on different types of sea-bottoms, from muddy to rocky, near offshore banks, on seamounts, and in cold-water reefs [16,17,18]. The vertical and bathymetric distribution of the species varies according to body size and season of the year: the larva is planktonic, whilst young individuals are found near the coasts with a major concentration in areas of relatively high productivity (e.g., river deltas) that are used as nurseries [14]. Young specimens and sub-adults are common in the shallower depths, down to a depth of 170 m, while the adults mainly inhabit the continental slope from 200 m to a depth of over 900 m [14,19,20]. This valuable deep-water fish has been worryingly exploited in the past decade, especially in the Bay of Biscay and Alboran Sea, where it is highly appreciated [17,18,21,22,23], and in Ionian and Aegean Seas [24]. At present, the Blackspot Seabream is considered to be overexploited in the Strait of Gibraltar [25]. Despite the commercial interest, the knowledge regarding the species’ ontogenetic cycle and how it affects the spatial dynamics of its populations remains poorly investigated. In general, the Blackspot Seabream exhibits protandric hermaphroditism, although a fraction of the population never changes sex because it is gonochoric [17,26]. Its peculiar biology and life-history traits, including slow growth rate, large size, late maturity, long life, and the mentioned sexual strategy, may lead the Blackspot Seabream to experience low recovery from overfishing [27]. Indeed, the yield is reduced because of premature capture before sexual maturation of females, resulting in a decrease in the census size of the populations [17]. For all these reasons, the current fishing pressure on this species is highly constrained by severe, although local, management measures (i.e., periodic closure of fishing activities, minimum size limits, gear restrictions, and Total Allowable Catch) in NE Atlantic waters [28,29]. In the Mediterranean Sea, the latest recommendation on the multiannual management plan for the sustainable exploitation of the Blackspot Seabream target fishery of the Gibraltar Strait (GSAs 1–3) was established in 2022 (GFCM/45/2022/3) and, among other measures, temporary closures have been suggested.

From a biological perspective, the current knowledge of the connectivity and differentiation of Blackspot Seabream populations derives from different techniques and separate approaches that have been progressively, but not yet holistically, integrated to properly assess its population structure at very different spatial scales [14,30]. The little available information on the dispersal capability of Blackspot Seabream was inferred according to comparisons with species having similar bio-ecological features [31,32,33]. To this end, Nadal et al. [30] studied and described how the Alboran Sea’s oceanographic features could passively control the dispersal of the Early Life Stage (ELS) individuals and lead to a secure connection between the Strait of Gibraltar and the Alboran Sea itself [34].

Previous studies applied molecular markers as mitochondrial DNA sequences, allozyme polymorphism, and available panels of nuclear microsatellite loci to assess the differentiation among samples from the North-Eastern Atlantic Ocean, the Azores Islands, and Mediterranean Sea locations [12,35,36,37,38,39]. These works underlined the overall lack of differentiation among scattered samples from the Mediterranean and Atlantic waters, and their separation from individuals from the isolated Azorean seamounts. Non-genetic studies on similar geographic samples were performed using fish morphometrics [39] and parasites as biological tags [40,41,42] to infer population structuring in the North-Eastern Atlantic Ocean and in the Mediterranean basin. Overall, morphometry highlighted the differentiation of individuals collected in the Southern Adriatic Sea from those from Portugal, Spain, and Greece [39], while the use of biological tagging based on ecto- and endoparasites (i.e., *Anisakis simplex*, *Bolbosoma* spp., and *Diphterostomum vividum*) revealed three main sub-populations of *P. bogaraveo* belonging to the Portuguese mainland from Porto to Sagres, Madeira, and the Azores, respectively [40], confirming previous genetic studies conducted in the Atlantic area [12,35,36,37,38,39].

With an unprecedented sampling design accomplished by EU and non-EU countries overlooking the Atlantic and Mediterranean coasts, covering most of the described distribution area of the species, we used 29 microsatellite loci to investigate the genetic variability of Blackspot Seabream over 320 specimens across 14 sampling locations and 6 macro-areas, from the Atlantic Ocean and the Strait of Gibraltar, to the Western, Central, and part of the Eastern Mediterranean Sea. The molecular data were analysed to assess genetic diversity and differentiation in *P. bogaraveo* individuals collected across the abovementioned wide geographical range. Thus, this study is an example of maximised efforts to obtain an exhaustive picture of the species’ genetic variability assessed in recent years.

The deepening of scientific knowledge, especially of biology, ecology, and behaviour of the targeted species, embodies a priority for understanding its role in the marine realm, its history across potential boundaries, and its response to local, regional, or wider scale variations. From this perspective, our final aim consisted of estimating the genetic diversity, the connectivity, and the potential migratory behaviour of adult Blackspot Seabream, and the dispersal of ELS individuals across six macro-areas, with a special focus on the Alboran Sea, where the species has been appreciated and highly exploited in past decades. We suggest and discuss different interpretations of the high homogeneity obtained, mainly related to the importance of egg and larval dispersal in contributing to the connectivity of the species on such a large geographical scale.

## 2. Materials and Methods

### 2.1. Sampling

**Figure 1:**
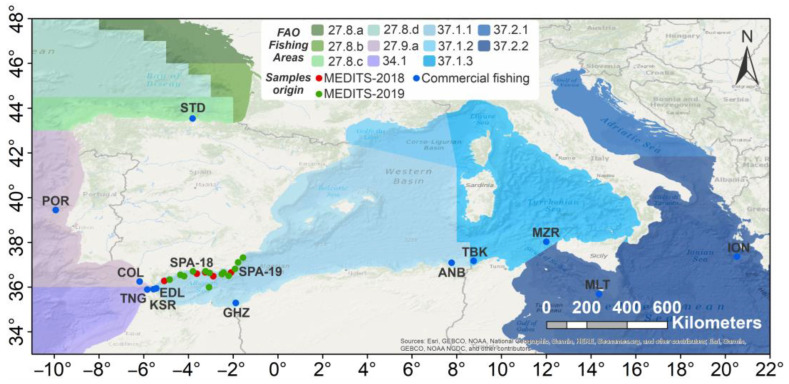
Map of geographical distribution of the sample analysed. Geographical coordinates are available only for the MEDITS-2018 (red dots) and MEDITS-2019 (green dots) samples. Refer to Table 1 for location codes. Location codes refer to landing ports. Sources (basemap): Esri, GEBCO, NOAA, National Geographic, DeLorme, HERE, Geonames.org, and other contributors. Maps were created using ArcMap™ (version 10.8) software of ArcGIS (https://www.arcgis.com/, accessed on 16 January 2023). ArcGIS^®^ and ArcMap™ are the intellectual property of Esri and are used herein under license.

A total of 320 individuals were collected during scientific surveys from the EU-funded MEDITS data collection [43] between 2018 and 2019 and from contracted commercial fisheries along the European and African coasts within the framework of the TRANSBORAN project activities [30], for a total of 14 sampling locations ascribable to six macro-areas (Table 1 and Figure 1). Where possible, sex and size measurements (i.e., weight and total length) of individuals were recorded (Table S1). Muscle tissue (about 1 cm^3^) was collected from fresh or frozen specimens and immediately preserved in 96% ethanol. The ID-labelled vials containing the tissue were stored at −20 °C until further processing.

### 2.2. Genetic Data Analysis

Detailed protocols used for DNA extraction, PCR amplification, and genotyping of nuclear markers [44,45,46] are described in the Supplementary Material Text S1.

### 2.3. Data Analysis

Raw data were imported into GeneMarker v.2.7.4 (SoftGenetics) and the peak calling was performed following the guidelines described in Guichoux et al. [47] and Flores-Rentería and Krohn [48]. A 10% threshold for missing data was set at both loci and individual levels.

GENEPOP v.4.2 [49,50] was used to evaluate the Linkage Disequilibrium among pairs of loci across all populations and the percentage of private alleles [Mean Frequency of Private Alleles p (1)]. Departures from Hardy–Weinberg equilibrium (HWE) for each locus and population were determined using Fisher’s (1935) exact test as implemented in the same software. The Markov Chain Monte Carlo (MCMC) approximation involved 10,000 dememorization steps, 1000 batches, and 10,000 iterations per batch. Probability tests were also conducted, and the relative values were corrected for multiple testing at alpha 0.05 using the Bonferroni correction.

The MICROCHECKER v.2.2 software [51] was used with default settings to infer the presence of null alleles (NA), PCR stuttering, and large allele drop-out. In parallel, a total of 1000 runs with FreeNA [52] were performed to determine the frequencies of null alleles across populations and loci, and to compute the F_ST_ values with or without the ENA (Excluding Null Alleles) correction.

Allele frequencies, mean number of alleles per locus, expected and observed heterozygosity values, and polymorphism index were calculated using the GENETIX software package v. 4.05 [53]. Allelic richness was estimated using FSTAT v. 2.9.3 [54]. The same software was used to calculate the coefficients of inbreeding (F_IS_).

The program ML-RELATE [55] was used to identify potential siblings among samples collected in the same location. Selecting the pairwise hypothesis testing option, the software calculates the likelihood ratio of relatedness between pairs of individuals and then estimates the probability of obtaining this ratio given the hypothesised relationship (Half or Full Siblings, HS and FS, respectively, or Parent–Offspring relationship) using a simulated null distribution based on observed population allele frequencies.

The statistical power for rejecting the null hypothesis of genetic homogeneity at various levels of F_ST_ was evaluated on the final dataset using the software POWSIM [56,57]. The sample sizes, number of loci, and allele frequencies obtained in this study were provided as an input to simulate sampling from a specified number of populations that have diverged to predefined levels of divergence (quantified as distribution of F_ST_).

Based on the results of descriptive analyses regarding the presence of NA and/or the departure from HWE, population differentiation tests and demography were investigated on a reduced dataset.

The pairwise F_ST_, following Weir and Cockerham’s model (1984), were calculated with ARLEQUIN v. 3.5 [58] with 20,000 permutations and alpha 0.05. The same software was used to test hierarchical genetic differentiation via analysis of molecular variance (AMOVA [59]), performed in different classes based on the results from pairwise F_ST_ analysis.

The adegenet and ade4 packages [60] for R software [61] were used to perform a Discriminant Analysis of Principal Components (DAPC) to identify and describe genetic clusters between two sample groupings (14 sampling locations and 6 macro-areas, Table 1) to infer the genetic structure of *P. bogaraveo* considering differently balanced sample sizes.

The same principle was followed to perform a Principal Coordinates Analysis (PCoA) to explore and visualise similarities or dissimilarities of data with GenAlEx v. 6.5 [62]. STRUCTURE [63] allowed the Bayesian clustering of individuals based on genotypes. To perform this analysis, a burning period of 50,000 iterations followed by 500,000 Markov Chain Monte Carlo (MCMC) replications, using the admixture model, Sampling Location Information as a prior (LOCPRIOR) parameter, and alpha = 1 were set, and the Allele Frequencies Correlated option was selected. To verify the consistency of responses across runs, ten runs for each given K (1–14) were computed. The evaluation of the best K was performed according to the Pritchard criterion [63] through the web-based STRUCTURE HARVESTER [64]. The web based CLUMPAK [65] was used to display the presented bar plots.

The Wilcoxon sign-rank test for heterozygosity excess [66] was applied using BOTTLENECK [67] to detect recent bottleneck events, under the Two-Phase Model (TPM) with 10,000 iterations and 95% single-step mutations. The qualitative descriptor of the allele frequency distribution known as the “mode-shift” indicator was also employed to assess distortions in allele frequency, as an indication of possible bottlenecks. In general, in a population near mutation-drift equilibrium, there is almost an equal probability that a locus will show a low heterozygosity excess or a heterozygosity deficit. On the contrary, when a bottleneck event is recent, most loci should exhibit an excess of heterozygosity.

## 3. Results

### 3.1. Genetic Diversity

Over the 29 microsatellite loci tested, the cross-amplification of the seven loci isolated in *Pagellus erythrinus* failed in *Pagellus bogaraveo* (showing no amplification, mismatched annealing, or monomorphism), leading to the exclusion of those markers from subsequent analyses, together with the species-specific Pb-OVI-D101 locus (no amplification obtained). Similarly, the locus PbMS17 was excluded because of inconsistent peak calling. The test for Linkage Disequilibrium did not show any significant results after Bonferroni correction, so no further loci were excluded at this stage.

With the exclusion of individuals showing 10% of missing data across the remaining 20 loci, the final dataset used for further analyses counted a total of 309 individuals. The percentage of private alleles [Mean Frequency of Private Alleles p (1)] calculated on the dataset thus defined corresponded to 3.1% and did not concern any specific location.

Significant deviations from HWE, after Bonferroni correction, were observed in all locations (Table S3) and in 13 of 20 microsatellite loci (Table 2).

MICROCHECKER software indicated the presence of Null Alleles (NA), as reflected in the departure from HWE observed across loci. The errors for stuttering (ST) were detected only in PbMS20 for STD, POR, ANB, MLT, and ION locations, in PbMS18 for STD, POR, and TNG locations, and in Pb-OVI-D114 for the KSR location. No evidence of large allele dropout was detected.

The number of individuals for each location, the expected (Hexp) and observed heterozygosity (Hobs), mean values of the number of alleles, allelic richness, FIS values, and HWE results for geographical location are summarised in Table S3, while the same statistics are reported for each microsatellite locus in Table 2. Overall, Hobs did not vary significantly across the sampling locations and ranged between 0.62 in KSR and 0.75 in MLT and ION (Table S3); this showed a greater range when considering the reduced dataset of 14 loci not affected by NA (see details below), ranging between 0.46 in Pb-OVI-D108 or in PbMS1 and 0.90 in PbMS4 (Table 2). In both set of markers considered (20 or 14 loci), this measure pinpointed a heterozygote deficiency when compared with the Hexp and the significantly different from zero and positive FIS values at all the sampling locations (ranging from 0.12 to 0.26) and over all loci (ranging from 0 to 0.51, except for Pb-OVI-D20 and Pb-OVI-D21).

After testing for relatedness between individuals captured in the same location, 92.92% of the individuals were unrelated, while 6.38% and 0.05% resulted in Half and Full Sibling relationships, respectively, but regardless of the geographical origin. No Parent–Offspring correlation was observed across the dataset.

The mean value of allelic richness was normalised using the minimum number of individuals within a location (nine individuals for GHZ). Sample SPA-19 showed the highest value of mean allelic richness (8.87), while TNG showed the lowest (7.95). However, the results indicated high levels of genetic diversity, with an average of 22.20 alleles per locus. The mean number of alleles per locus estimated in each location fluctuated between 15.80 and 8.70 in SPA-19 and GHZ locations, respectively. The results appeared to be related to the sample size, since allelic richness and expected heterozygosity were similar for all locations (Figure S1).

Analyses of statistical power conducted with POWSIM indicated that the complete marker set (20 loci) and that using 14 loci (see below) detected F_ST_ = 0.005 or higher values, with a probability close to one, for sample sizes and allele frequencies obtained from the samples in this study. At low levels of divergence, 20 microsatellites are more powerful for detecting structuring than 14 microsatellite loci (Table S4). The chi-square and Fisher approach α-errors are close to 5% for both marker sets, even if the Fisher approach appears overly conservative with an α error of only 0.46–0.82%.

However, considering the results of descriptive analyses of genetic diversity, six markers were excluded since NA and deviations from the HWE characterised more than 50% of the 14 sampling locations. Thus, the following analyses focussing on population differentiation and demography were performed on a reduced dataset including 309 individuals and 14 microsatellite loci.

### 3.2. Population Differentiation and Demography

When considering pairwise comparisons of F_ST_ values between sampling locations, low estimates of this index were highlighted across the dataset (F_ST_ ranging between 0.000 and 0.050). Nevertheless, a pattern of genetic differentiation was mainly observed when comparing ANB with STD, KSR, SPA-19, TBK, MZR, and ION (Table 3). Low but significant values of F_ST_ also involved the comparison of TBK with COL, KSR, TNG, SPA-19, ANB, and MZR (Table 3). Moreover, GHZ was found to be significantly different from COL (F_ST_ 0.012; Table 3).

Consistent with these findings, three scenarios were used to perform the AMOVA (Table 4):

Scenario 1: One Group, no geographical differentiation.

Scenario 2: Six Groups—Group 1: STD; Group 2: POR; Group 3: COL, KSR, TNG, EDL; Group 4: SPA-18, SPA-19, GHZ; Group 5: ANB, TBK; Group 6: MZR, MLT; Group 7: ION.

Scenario 3: Two Groups—Group 1: STD, POR, KSR, TNG, EDL, COL, SPA-18, SPA-19, MLT, MZR, ION; Group 2: GHZ, TBK, ANB.

A low but significant (alpha 0.05) percentage of variation was observed among groups in AMOVA performed for scenario 3. The percentage of variation among locations within groups ranged from 0.10 to 5.17 and was significant for both scenarios 1 and 2. The largest variation was observed within individuals and ranged from 94.48 to 94.64 and was significant in all tested groupings (Table 4).

**Figure 2:**
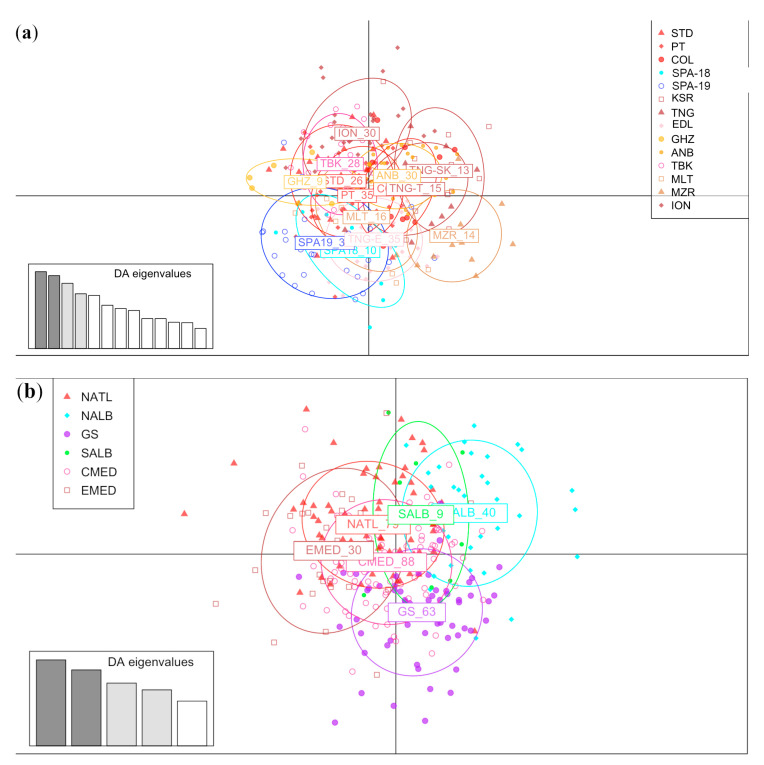
(**a**) Result of DAPC analysis performed on 14 locations. (**b**) Result of DAPC analysis performed on 6 macro-areas.

The results from DAPC analyses were obtained involving fourteen locations and six macro-areas (Figure 2a and Figure 2b, respectively). In both representations, the points were scattered, and no clear sign of differentiation was observed between locations.

The results from PCoA based on 14 locations did not reveal any genetic divergence on a geographical basis (Figure S2A). In contrast, when considering six macro-areas (Figure S2B), a slight differentiation was observed between three different groups composed of the samples from the Atlantic area (i.e., POR, STD, and COL), Northern Alboran Sea (i.e., SPA-18 and SPA-19), and Eastern Mediterranean (ION) compared to the samples from Western Gibraltar Strait (i.e., KSR, TNG, and EDL), Southern Alboran Sea (GHZ), and Central Mediterranean areas, respectively (i.e., ANB, TBK, MLT, MZR).

**Figure 3:**
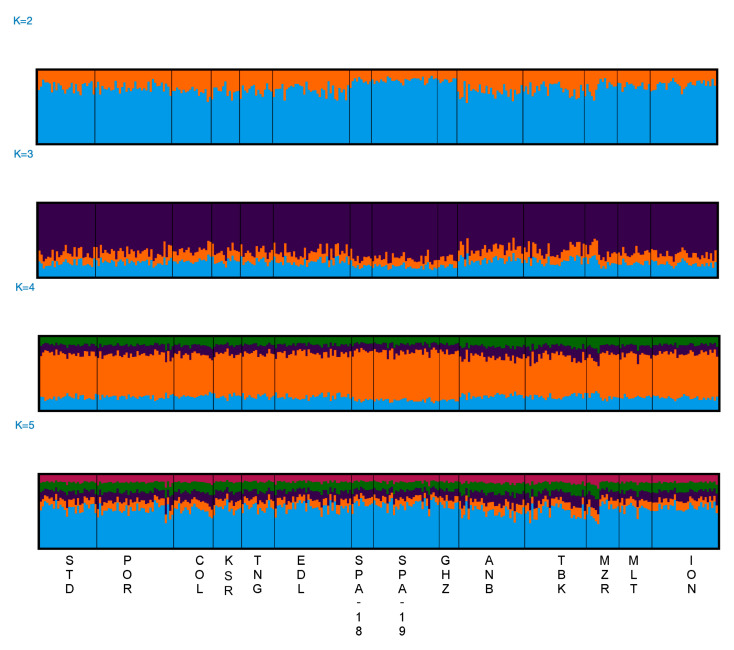
Summary plot of the STRUCTURE analysis from K = 2 to K = 5. Each individual is represented by a single vertical line on the x-axis. Coloured segments represent the membership of individuals in the population clusters. Results are shown for the 14 locations using 14 microsatellite loci.

The results from STRUCTURE were analysed using the LnP(D) trend to evaluate the best K according to STRUCTURE HARVESTER. The plot did not show the expected plateau usually displayed when a population structure is present (Figure S3). The plot showed an increase in LnP(D) variance between runs in relation to the increase in K (Figure S3). Individual bar plots grouped from K = 2 to K = 5 clusters (Figure 3) showed an admixed genetic component across all the investigated locations.

BOTTLENECK outcomes (Table S5) showed no evidence of significant deviation of expected heterozygosity Hexp (calculated from allele frequencies) to expected heterozygosity Heq at mutation drift equilibrium (calculated from the number of alleles and sample size); thus, no evidence of significant population bottlenecks was detected under any of the mutation models implemented. The only exception was the EDL population sample (*p*-value: 0.05) for tail 1, which showed a deficit of heterozygosity.

## 4. Discussion

Along with conservation and management purposes, the identification of fish stock units is also a major topic in exploited populations, and the inconsistency between the biological and management scale of the units’ boundaries can hamper effective fisheries management [68]. In this study, we assessed the genetic variation and population structure of a commercially valuable and overexploited species, *Pagellus bogaraveo*, by applying both complete and selected panels of microsatellite loci to geographical samples collected in the main fishing areas of the Atlantic Ocean and the Mediterranean Sea. Here, the choice of the nuclear markers to be applied underwent an experimental workflow that highlighted the failure of cross-amplification of nuclear loci originally isolated from *P. erythrinus* on *P. bogaraveo*. This failure may be linked to rapid divergence between congeneric species, leading to erroneous amplification of DNA fragments [69,70]. On the other hand, when assessing species-specific markers, no issue due to PCR artefacts affected our genotyping scoring, although multiplex amplification was performed [71,72].

The chosen methods for the assessment of Null Alleles (NA) frequencies showed that NA are present in the dataset overall, especially associated with loci isolated and characterised from individuals collected around the Azores Islands [44]. As a matter of fact, Stockley et al. [12] also indicated PBMS1 and PBMS2 loci as triggers of uniform heterozygote deficiency, displaying high frequencies of NA across all the targeted locations along the Portuguese coasts and Azores Islands. The same choice was adopted in Piñera et al. [37], where PB-OVI-A5 and PB-OVI-D101 were removed from subsequent analyses carried out on individuals collected in the North-Eastern Atlantic Ocean and the Northern Alboran Sea.

The genetic diversity of Blackspot Seabream populations in terms of the number of alleles per locus and per population, and the expected heterozygosity across loci, was consistent with that found in previous studies [12,37], although a higher number of alleles was observed in this work. The most polymorphic locus according to Stockley et al. [44], PBMS16, showed 18 alleles, while 42 alleles were found in the present paper. The same result involved the nuclear markers developed by Piñera et al. [37], in which the number of alleles ranged between 5 (Pb-OVI-D108) and 35 (Pb-OVI-A5). Here, those markers showed 7 and 41 alleles, respectively. This evidence could be related to the higher number of samples and individuals analysed here, as highlighted by the positive correlation between the mean number of alleles and the sample size per population (see Figure S1).

Despite high F_IS_ values (0.122–0.263) over all loci, HWE deviations were not persistent, and the relatedness test conducted across sampling locations did not highlight sibling relations. Most of all, it confirmed the total absence of co-occurrence of parents and offspring in the analysed samples. A low percentage of HS and FS confirmed the lack of inbreeding inside the tested populations.

Many of the markers included in this study have already proved to be prone to NA flaws but, to date, there is no consensus about the appropriate limit of NA to be allowed across loci and geographical samples [73,74,75]. Furthermore, the results obtained with POWSIM confirmed the high-resolution power of the dataset built on both 20 and 14 microsatellite loci when an F_ST_ = 0.005 or higher was considered, leading to the conclusion that no informative loci were excluded from the dataset when using the restricted dataset (14 microsatellite loci) in the analyses of genetic differentiation.

Regarding the genetic population structure, results supported the lack of genetic boundaries at both the geographical locations and macro-area levels, revealing a continuum in the gene flow of this deep-water species across the Western-Central Mediterranean Sea and adjacent Atlantic coasts. According to AMOVA (Table 4), the highest variation was observed at the individual level, likely reflecting the high genetic diversity values found in and consistent with the percentage of variation measured by Stockley et al. [12], which ranged from 87.88% in the D-loop fragment (mtDNA) to 99.64% in nuclear loci. A high percentage of variation within the population class (99.89%) and low variation among regions (0.19%) were observed by Piñera et al. [37] on a similar, but restricted, geographical scale. The DAPC plot coherently showed a scattered pattern, confirming the lack of genetic differentiation between investigated populations and the significant variability within samples (Figure 2A,B). In the analysis performed using six macro-areas, only the Southern Alboran Sea (SALB, corresponding to the GHZ sampling location), seemed to be more differentiated, but this group was characterised by a very limited sample size. This small differentiation was confirmed by the PCoA performed on six macro-areas, while high similarity was observed between the samples from the Atlantic area and the Eastern Mediterranean Sea. This result was also observed when estimating low, but significant, pairwise F_ST_ values (Table 3), characterising the geographical samples collected from the Southern Alboran Sea (Algeria) and the Gulf of Tunis and from the neighbouring Western Sea of Sicily (GHZ, ANB, TBK, and MZR).

Individual-based approaches revealed a homogeneous distribution of genetic components across sampling locations, confirming the lack of distinct clusters for *P. bogaraveo*. Despite the application of a considerable panel of highly polymorphic nuclear markers, coupled with a large sample size covering large distances, our study did not reveal genetic differentiation signals in full agreement with previous studies. This evidence could be explained by biology and life-history traits of *P. bogaraveo*, but dispersal capability of egg and larval stages during the drifting phases (i.e., timing, depth preference, and development time) and adults’ reproductive strategies (i.e., fecundity and spawning migrations across several regions) remain poorly investigated. Indeed, studies of adults’ residency and migratory behaviour of young specimens at a regional level could identify feeding grounds for sub-adult fish and spawning grounds adopted by mature individuals. Evidence produced to date highlighted that Blackspot Seabream individuals live in small shoals mainly composed of adults dwelling near the sea-bottom, where they are prevalently resident [14,16,17,19]. Short migrations were documented from coastal to offshore areas by mark–recapture data, and limited movements were observed around the same islands or seamounts [18,26,28,74], characterised by seasonal increases in food availability driven by upwelling [76,77].

Although a general agreement exists regarding the Strait of Gibraltar as the main spawning area for the Eastern Atlantic Ocean–Western Mediterranean region [14,33,78], scarce information is available about daily and seasonal displacements of adults, spawning strategies (especially depth range), and ELS dynamics (buoyancy, Pelagic Larval Duration, etc.). Nevertheless, the migration of spawners registered by otoliths’ microchemistry [34], and the powerful tidal dynamics of the Strait of Gibraltar, strongly affect the dispersal patterns of the ELS and may justify the genetic homogeneity observed across the Alboran region. The general dispersal pattern of ELS seems to interest the northern (Tarifa) and southern Strait (Tangier), and it is strongly modulated by distance, travelling speed, and scattering by the fortnightly tidal cycle (spring–neap tide alternation) and spawning depth [33,78,79]. Here, the Atlantic jet might be the main cause of a zonal (west–east) connectivity within the Alboran Sea, while the north–south and less stable dispersal might depend on the low-frequency variability of the flow [33]. Such segregation and tri-dimensional distribution might then help the different life stages to bypass potential oceanographic barriers.

The potential mobility of adults related to spawning events and the segregation between the typical habitat of juveniles and adult specimens has also been demonstrated through the morphological and microchemical variations in the otoliths of Hidalgo et al. [34,80]. When considering the otolith’s core collected from the Atlantic Ocean and Mediterranean Sea, Ref. [34] described a considerable overlap between samples, suggesting a geographical common origin of the Blackspot Seabream (i.e., common spawning ground and seasonal migration). Conversely, and with no support of genetic evidence, information retrieved from the edge of the otoliths, which can capture the chemical composition of the adult dwelling environment, discriminated adult specimens of Blackspot Seabream inside the Central and Eastern Mediterranean Sea (FAO areas 37.1.3 and 37.2.2) from those inhabiting other areas, pointing to a potential spawning migration operating in the Alboran Sea [34].

In terms of evolutionary history, recent studies estimated that the paleoclimatic crisis that occurred in the early Pleistocene may have led to the divergence between Atlantic and Mediterranean populations of *P. bogaraveo* and the isolation of the Azores archipelago as a glacial refugia [40,81]. A more recent and independent re-colonisation of the Mediterranean Sea from the Atlantic Ocean could have happened soon after a strong bottleneck in Mediterranean populations [81,82,83,84]. Nevertheless, dedicated tests conducted here did not show evidence of significant population bottlenecks under any of the mutation models implemented (Table S5). If any restriction to *P. bogaraveo* gene flow is present between Mediterranean and Atlantic areas, it should be sought by intensifying the sampling effort along the eastern Atlantic Ocean across a larger investigation scale (e.g., from Cape Blanc to Norway, corresponding to the southernmost and northernmost limits of the species’ distribution [12,13,14]). Furthermore, microsatellite mutation rates are unknown for this species, leading to erroneous estimations of effective population size (Ne) in relation to the fitness-dependent census size [85,86,87,88]. Given the lack of this information, more exhaustive investigations of the biology of Blackspot Seabream, particularly regarding its eggs and larval dispersal by drift and adult migrations, should be conducted in the future. Moreover, the use of DNA sequences, on one hand [38] and Single Nucleotide Polymorphisms (SNPs), on the other, would produce new data to be compared with publicly available records (i.e., the mitochondrial DNA Control Region from other geographical locations) and enable the discovery of new genetic tags to deepen the investigation of the population structure and connectivity patterns. The different methodologies reported here underlined that the most efficient approach to studying the population structure of a species is multidisciplinary, which would allow the comparison and integration of the results from different methods, considering their relative strengths and weaknesses. Given the multidimensional nature of the stock concept for fishery purposes [89], new studies integrating previous evidence on morphometry, parasite communities, and otolith microchemistry could identify significant differences between Atlantic and Mediterranean samples [39,40,82] and effectively contribute to the resolution of the bigger picture depicting the stock boundaries and status of such an important resource as the Blackspot Seabream.

## 5. Conclusions

Based on an extensive sampling effort targeting most of the geographical distribution of the Blackspot Seabream, the species’ genetic variability and its population connectivity were estimated using available, powerful markers and multiple analytical approaches. The results described here contribute to the bigger picture of the overall variability, characterising even distant population samples of *P. bogaraveo*, and showed the species’ overall genetic homogeneity, which is likely related to its dispersal potential across different life stages. Because of the limitations related to the applied genetic markers, the testing of more informative genome-wide markers is recommended to identify hidden genetic structures and update effective management measures for the responsible use of this important and appreciated fishery resource.

## Figures and Tables

**Table 1:** Details of sampling locations and relative codes, with reference to the FAO major fishing area, FAO zone, Regional Sea, the number of collected samples (N), and the period of sampling. Location refers to landing ports when the information was available. N. a. is reported when sampling location/port’s name was not available.

FAO Fishing Area	FAO Zone	Regional Sea	Country	Location Name	Macro-Area/Macro-Area Code	Location Code	N
27.8.c	Bay of Biscay—South	Atlantic Ocean	Spain	Santander	Atlantic Ocean NATL	STD	26
27.9.a			Portugal	N. a.		POR	36
27.9.a			Spain	Conil		COL	18
27.9.a	Portuguese Waters—East	Atlantic Ocean/Alboran Sea	Morocco	Ksar Sghir	Western Gibraltar Strait GS	KSR	15
27.9.a			Morocco	Tangier		TNG	18
27.9.a			Morocco	Eddalya		EDL	35
37.01.01	Western Mediterranean—Balearic	Alboran Sea	Spain	MEDITS 2018	Northern Alboran Sea NALB	SPA-18	10
37.01.01			Spain	MEDITS 2019		SPA-19	30
37.01.01		Alboran Sea/Balearic Sea	Algeria	Ghazaouet	Southern Alboran Sea SALB	GHZ	9
37.01.01		Balearic Sea	Algeria	Annaba	Central Mediterranean Sea CMED	ANB	30
37.01.01		Balearic Sea/Tyrrhenian Sea	Tunisia	Tabarka		TBK	30
37.01.03	Western Mediterranean—Sardinia	Sea of Sicily	Italy	Marettimo		MZR	16
37.02.02	Central Mediterranean—Ionian	Sea of Sicily	Malta	N. a.		MLT	17
37.02.02		Ionian Sea	Greece	N. a.	Eastern Mediterranean Sea EMED	ION	30
Total N							320

**Table 2:** Genetic diversity at 20 microsatellite loci over 14 *Pagellus bogaraveo* geographic locations. Number of alleles, Hexp: expected heterozygosity; Hobs: observed heterozygosity, Mean AR: mean allelic richness, F_IS_: inbreeding coefficient, HWE: Hardy–Weinberg equilibrium; in bold, significant results after Bonferroni correction (alpha: 0.0001786).

Locus	N. Alleles	Hexp	Hobs	Mean AR	F_IS_	HWE
Pb-OVI-B2	23	0.86	0.82	8.65	0.07	0.004119
PbMS2	20	0.85	0.56	8.38	0.37	**<0.00 × 10^0^**
PbMS6	37	0.94	0.87	12.39	0.10	**<4.91 × 10^−12^**
PbMS16	42	0.94	0.86	12.56	0.11	**<6.52 × 10^−15^**
PbMS19	10	0.67	0.59	4.24	0.15	**<9.43 × 10^−7^**
Pb-OVI-D108	7	0.46	0.46	2.89	0.00	**7.27 × 10^−6^**
Pb-OVI-A5	41	0.91	0.83	10.95	0.13	**<5.30 × 10^−11^**
Pb-OVI-D114	11	0.81	0.74	6.36	0.12	0.00568
Pb-OVI-D102	20	0.88	0.72	8.86	0.21	**<6.98 × 10^−13^**
Pb-OVI-D20	12	0.82	0.86	6.70	−0.03	0.790455
Pb-OVI-D21	14	0.84	0.87	7.38	0.00	0.978329
Pb-OVI-D106	11	0.79	0.81	5.75	0.00	0.647982
Pb-OVI-A3	22	0.88	0.71	9.16	0.22	**<2.32 × 10^−15^**
PbMS20	21	0.83	0.45	7.32	0.49	**<0.00 × 10^0^**
PbMS18	23	0.83	0.50	8.37	0.42	**<0.00 × 10^0^**
PbMS1	38	0.92	0.46	11.04	0.52	**<0.00 × 10^0^**
Pb-OVI-D22	12	0.82	0.83	6.89	0.02	0.568561
PbMS4	29	0.91	0.90	10.30	0.04	**<1.18 × 10^−5^**
PbMS15	40	0.92	0.74	11.70	0.23	**<3.24 × 10^−31^**
Pb-OVI-C103	11	0.76	0.72	5.53	0.08	0.02496

**Table 3:** Pairwise F_ST_ values estimated in 14 geographic locations and 14 loci. *p*-values are reported above the diagonal. In bold, significant results at alpha: 0.05.

	STD	POR	COL	KSR	TNG	EDL	SPA-18	SPA-19	GHZ	ANB	TBK	MZR	MLT	ION
STD		0.926	0.915	0.248	0.117	0.639	0.358	0.700	0.206	**0.021**	0.304	0.866	0.340	0.635
POR	−0.003		0.706	0.176	0.595	0.84	0.792	0.553	0.608	0.197	0.503	0.444	0.779	0.874
COL	−0.004	−0.001		0.139	0.131	0.342	0.361	0.194	**0.050**	0.159	**0.036**	0.870	0.350	0.123
KSR	0.006	0.006	0.009		0.641	0.344	0.091	0.075	0.196	**0.024**	**0.015**	0.731	0.394	0.307
TNG	0.007	0.000	0.006	0.002		0.89	0.743	0.070	0.213	0.530	**0.048**	0.156	0.255	0.390
EDL	0.000	−0.001	0.002	0.004	−0.002		0.693	0.395	0.163	0.189	0.104	0.499	0.435	0.439
SPA-18	0.003	−0.002	0.002	0.015	−0.002	−0.001		0.477	0.059	0.073	0.065	0.330	0.300	0.173
SPA-19	0.000	0.001	0.004	0.010	0.008	0.002	0.002		0.569	**0.001**	**0.044**	0.590	0.297	0.172
GHZ	0.007	0.000	**0.012**	0.013	0.008	0.007	0.015	0.001		0.644	0.454	0.066	0.434	0.201
ANB	**0.007**	0.003	0.004	**0.011**	0.001	0.003	0.008	**0.011**	−0.001		**0.001**	**0.004**	0.065	**0.019**
TBK	0.002	0.001	**0.008**	**0.014**	**0.008**	0.004	0.009	**0.006**	0.002	**0.010**		**0.041**	0.423	0.088
MZR	−0.003	0.002	−0.004	0.000	0.008	0.002	0.004	0.001	0.015	**0.014**	**0.009**		0.322	0.514
MLT	0.002	−0.002	0.001	0.004	0.004	0.001	0.003	0.003	0.001	0.006	0.001	0.003		0.122
ION	0.000	−0.002	0.004	0.004	0.002	0.001	0.005	0.003	0.005	**0.006**	0.004	0.000	0.004	

**Table 4:** Proportion of molecular variance among groups, among populations, and within individuals resulting from AMOVA performed on 14 geographic locations and 14 loci with three different groupings. In bold, significant results at alpha: 0.05.

Source of Variation	% of Variation	F Statistics		*p*-Value
Scenario 1				
Among population	0.20	F_ST_	0.002	1.00
Among individuals within population	5.17	F_IS_	0.052	**0.000**
Within individuals	94.63	F_IT_	0.054	**0.000**
Scenario 2				
Among groups	−0.12	F_CT_	−0.001	0.815
Among population within groups	0.31	F_SC_	0.003	**0.037**
Among individuals within population	5.17	F_IS_	0.052	**0.000**
Within individuals	94.64	F_IT_	0.054	**0.000**
Scenario 3				
Among groups	0.26	F_CT_	0.003	**0.014**
Among population within groups	0.10	F_SC_	0.001	0.162
Among individuals Within population	5.16	F_IS_	0.052	**0.000**
Within individuals	94.48	F_IT_	0.055	**0.000**

## Data Availability

The microsatellite data that support the findings of this study are available as a genotype matrix from the corresponding author upon request.

## Supplementary Materials

The following supporting information can be downloaded at: https://www.mdpi.com/article/10.3390/ani13172691/s1, Text S1: Molecular methods; Table S1: List of all the individuals included in the study. Associated data are reported when available: date of sampling, sex (M, male; F, female; H, hermaphrodite; HF, hermaphrodite/female; IND: indeterminate), latitude and longitude in decimal format, sampling point (“Verified” for the exact sampling point; “Not Verified” for arbitrarily chosen sampling point), Depth of sampling and sampling location; Table S2: Mix setup containing the 29 *P. bogaraveo* and the 7 *P. erythrinus* microsatellite loci. The first four mixes contained only *P. bogaraveo*’s microsatellite loci and the last two contained the microsatellite loci developed for *P. erythrinus*; Table S3: Genetic diversity at 14 geographic locations for all 20 loci. N. of individuals: number of analysed samples within a population; Hexp: expected heterozygosity; Hobs: observed heterozygosity. Mean number of alleles for each population. mean AR: mean allelic richness; F_IS_: inbreeding coefficient; HWE: Hardy–Weinberg Equilibrium: significant result after Bonferroni correction (alpha: 0.0001786); Table S4: Statistical power of different marker sets for detecting various levels of population differentiation (F_ST_) by both chi-square and Fisher’s method when using sample size and allele frequencies obtained by the samples in this study. Performed with POWSIM [55]; Table S5: Results from BOTTLENECK software [67]; in bold, significant results at alpha: 0.05; Figure S1: Rarefaction curve representative of the mean number of alleles and sampling units for 14 geographic locations; Figure S2: (a) Result from PCoA analysis performed on 14 locations with 14 microsatellite loci; (b) Result from PCoA analysis performed on 6 macro-areas with 14 microsatellite loci; Figure S3: LnP(K) graph resulting from STRUCTURE HARVESTER [64]. References [37,44,45,46] are mentioned in the Supplementary Materials.
