# Supplementary material for: Matching an Old Marine Paradigm: Limitless Connectivity in a Deep-Water Fish over a Large Distance
Source: Animals (Basel). 2023 Aug 23;13(17):2691. doi: 10.3390/ani13172691 (PMC10486518; doi:10.3390/ani13172691)
Supplement: Supplementary file 1 [file animals-13-02691-s001.zip › Suppl Text S1_Pagellus_Ferrari et al_rev22-08-2023.pdf]

Matching an Old Marine Paradigm: Limitless Connectivity in a Deep-Water Fish over a Large Distance

Alice Ferrari [1,*,†], Martina Spiga [1,†], Miriam Dominguez Rodriguez [2], Fabio Fiorentino [3,4], Juan Gil-Herrera [5], Pilar Hernandez [6], Manuel Hidalgo [7], Carolina Johnstone [2], Sana Khemiri [8], Kenza Mokhtar-Jamaï [9], Irene Nadal [10], Montse Pérez [11], Simone Sammartino [12], Marcelo Vasconcellos [13] and Alessia Cariani [1]

1 Department of Biological, Geological & Environmental Sciences (BiGeA), University of Bologna, 40126 Bologna, Italy; martina.spiga5@unibo.it (M.S.); alessia.cariani@unibo.it (A.C.)
2 Centro Oceanográfico de Málaga (IEO, CSIC), 29640 Málaga, Spain; mdr8625@gmail.com (M.D.R.); carolina.johnstone@ieo.csic.es (C.J.)
3 Institute for Marine Biological Resources and Biotechnology (IRBIM), National Research Council (CNR), 91026 Trapani, Italy; fabio.fiorentino@irbim.cnr.it
4 Stazione Zoologica Anton Dorhn, 90149 Palermo, Italy
5 Centro Oceanográfico de Cádiz (IEO, CSIC), 11006 Cádiz, Spain; juan.gil@ieo.csic.es
6 Technical Unit for Western Mediterranean, General Fisheries Commission for the Mediterranean (GFCM), Fisheries and Aquaculture Division, Food and Agricultural Organization (FAO) of the United Nations, 29014 Malaga, Spain; pilar.hernandez@fao.org
7 Oceanographic Center of the Balearic Islands, Ecosystem Oceanography Group (GRECO), Spanish Institute of Oceanography (IEO, CSIC), 07015 Palma, Spain; jm.hidalgo@ieo.csic.es
8 Institut National des Sciences et Technologies de la Mer, Salammbô 2025, Tunisia; sanak182000@yahoo.com
9 Laboratoire de Génétique des Populations Halieutiques, Institut National de Recherche Halieutique (INRH), Centre Régional d'Agadir, Agadir 80000, Morocco; mokhtarjamai@inrh.ma
10 Physical Oceanography Group, Instituto de Biotecnología y Desarrollo Azul (IBYDA), Universidad de Málaga, 29071 Málaga, Spain; irenenadal@ctima.uma.es
11 AquaCOV, Centro Oceanográfico de Vigo (IEO, CSIC), 36390 Pontevedra, Spain; montse.perez@ieo.csic.es
12 Physical Oceanography Group, Instituto de Ingeniería Oceánica (IIO), Universidad de Málaga, 29071 Málaga, Spain; ssammartino@ctima.uma.es
13 Fisheries and Aquaculture Division, Food and Agriculture Organization (FAO) of the United Nations, 00153 Rome, Italy; marcelo.vasconcellos@fao.org
* Correspondence: alice.ferrari6@unibo.it
† These authors contributed equally to this work.

Supplementary Material

Text S1

Molecular methods

DNA isolation

Before the DNA extraction, muscle tissues were treated adding 600 µL of ASL buffer (Qiagen) and about 50-100 µL of zirconium beads (Zirconia/Silica Beads, 0.1 mm). Samples were vortexed using the TissueLyser (Qiagen) for 1 min at 25 Hz followed by 1 min at 25 Hz and centrifuged at 14,000 g for 2 min. A total of 200 µL of supernatant was transferred in the S-block well for genomic DNA extraction. Total genomic DNA was obtained with the cador® Qiacube pathogen kit for QIAcube® HT System (Qiagen) and its quality was visualised on a 1.5% agarose gel electrophoresis. Failures in DNA extractions were followed by further attempts, using an improved protocol, in which approximately 0.02 g of tissue were homogenised in 100 µL of lysis buffer and the DNA IQ™ System-Database Protocol (Promega) was applied.

Nuclear markers and amplification conditions

A total of 22 microsatellite loci previously isolated and characterised from *Pagellus bogaraveo* [44,45] were amplified on the collected samples. Furthermore, we attempted the

cross-amplification of seven loci isolated and characterised from *Pagellus erythrinus* (Linnaeus, 1758) by Ramšak et al. [46]. In total, six different multilocus reactions were designed based on loci annealing temperature and expected amplicon size (Table S2). All multiplex PCR amplifications were performed using the Platinum® Multiplex PCR Master Mix (Thermofisher Scientific) in a total volume of 15 μL, with 7.5 μL of Multiplex master mix, 1.5 μL of enhancer and 1.1 μL of DNA. The amplification conditions included an initial denaturation at 95°C for 2 min, a denaturation at 95°C for 30 secs, an elongation at 60-62°C (depending on the mix) for 30 secs, a second denaturation at 95°C for 30 secs, an elongation at 68°C for 45 secs for a total of 32 cycles and a final extension step at 68°C for 30 secs. The separation of PCR products of individual samples was carried out on a 2.5% agarose gel electrophoresis. The fluorescently labelled DNA fragments carrying NED, FAM, PET, VIC and LIZ fluorochromes, were mixed with the GeneScan 500 LIZ (Applied Biosystems®) internal size standard and separated using the Applied Biosystems® 3130xl Genetic Analyzer. Technical replicates were included to estimate repeatability and error rates resulting from PCR amplification (assessing congruence of results between single locus and multiplex approaches) and fragment analysis. Replicates were issued from the same DNA source but were processed independently.

References

44. Stockley, B.M.; Rogers, A.D.; Iyengar, A.; Menezes, G; Santos, R.; Long, A. Ten microsatellite loci isolated and developed for the blackspot seabream, *Pagellus bogaraveo* (Brunnich 1768). *Mol. Ecol.* **2000**, *9*, 999–1000.
45. Piñera, J.A.; Bernardo, D.; Blanco, G.; Vazquez, E.; Sanchez, J.A. Isolation and characterization of polymorphic microsatellite markers in *Pagellus bogaraveo*, and cross-species amplification in *Sparus aurata* and *Dicentrarchus labrax*. *Mol. Ecol. Notes* **2006**, *6*, 33–35.
46. Ramšak, A.; Garoia, F.; Guarniero, I.; Mannini, P.; Tinti, F. Novel polymorphic microsatellite markers for the common pandora (*Pagellus erythrinus*). *Mol. Ecol. Notes* **2003**, *3*, 553–555.
