# Supplementary figures and images for: Matching an Old Marine Paradigm: Limitless Connectivity in a Deep-Water Fish over a Large Distance
Source: Animals (Basel). 2023 Aug 23;13(17):2691. doi: 10.3390/ani13172691 (PMC10486518; doi:10.3390/ani13172691)

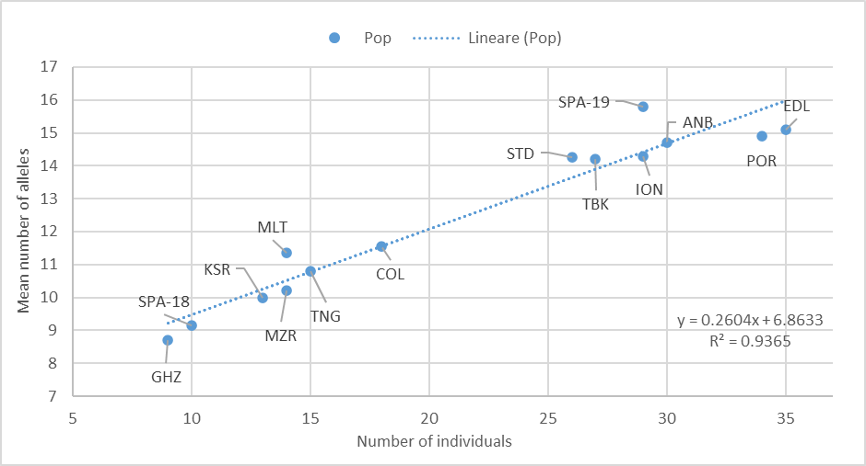

Supplement: Supplementary file 1 [file animals-13-02691-s001.zip › Figure S1.png]

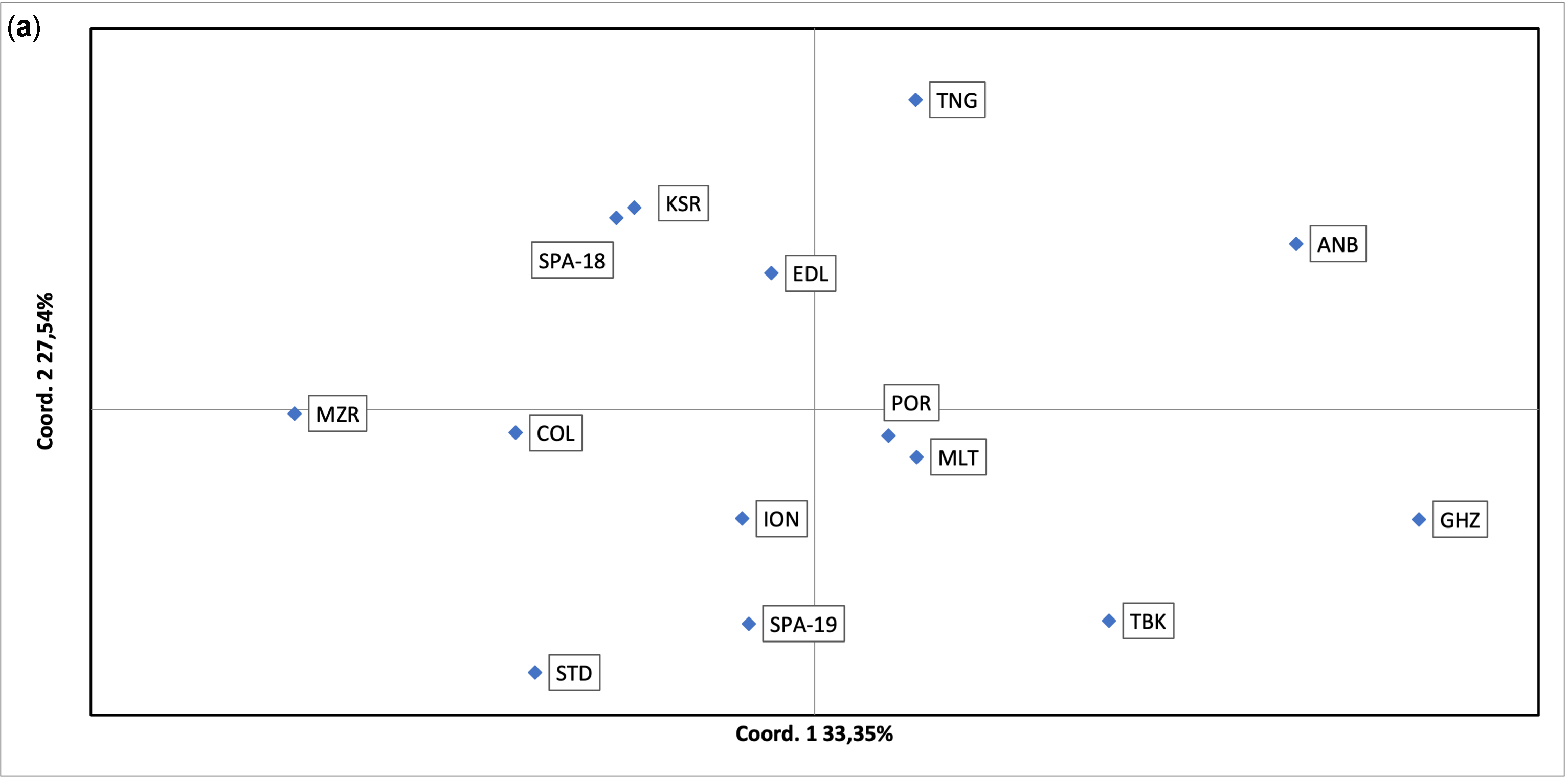

Supplement: Supplementary file 1 [file animals-13-02691-s001.zip › Figure S2a.JPEG]

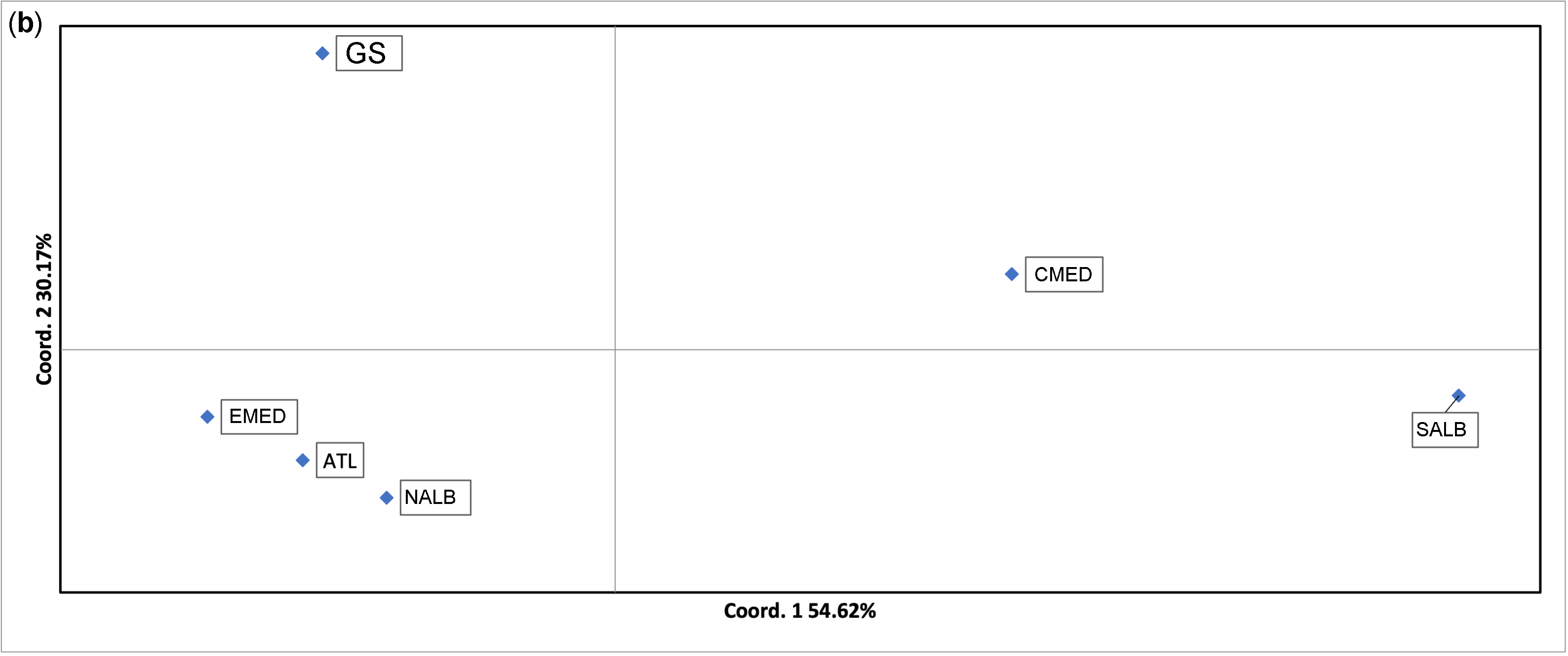

Supplement: Supplementary file 1 [file animals-13-02691-s001.zip › Figure S2b.JPEG]

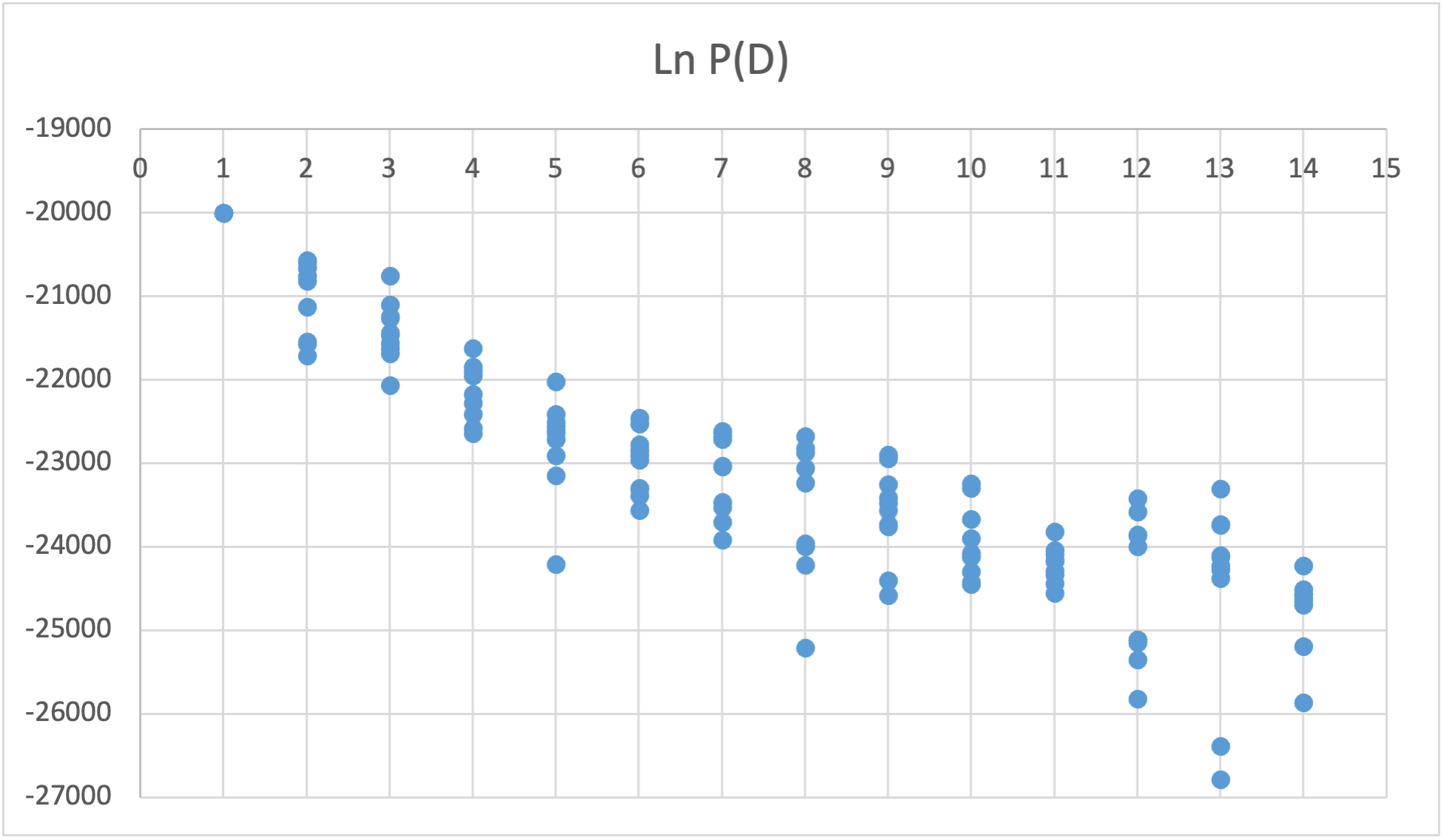

Supplement: Supplementary file 1 [file animals-13-02691-s001.zip › Figure S3.JPEG]
